# Insights into the Role of Membrane Lipids in the Structure, Function and Regulation of Integral Membrane Proteins

**DOI:** 10.3390/ijms22169026

**Published:** 2021-08-21

**Authors:** Kenta Renard, Bernadette Byrne

**Affiliations:** Department of Life Sciences, Imperial College London, Exhibition Road, London SW7 2AZ, UK; kenta.renard18@imperial.ac.uk

**Keywords:** integral membrane protein, membrane lipid, structure, function, oligomeric state, cryo-EM, advanced mass spectrometry, molecular dynamics, membrane mimetic systems

## Abstract

Membrane proteins exist within the highly hydrophobic membranes surrounding cells and organelles, playing key roles in cellular function. It is becoming increasingly clear that the membrane does not just act as an appropriate environment for these proteins, but that the lipids that make up these membranes are essential for membrane protein structure and function. Recent technological advances in cryogenic electron microscopy and in advanced mass spectrometry methods, as well as the development of alternative membrane mimetic systems, have allowed experimental study of membrane protein–lipid complexes. These have been complemented by computational approaches, exploiting the ability of Molecular Dynamics simulations to allow exploration of membrane protein conformational changes in membranes with a defined lipid content. These studies have revealed the importance of lipids in stabilising the oligomeric forms of membrane proteins, mediating protein–protein interactions, maintaining a specific conformational state of a membrane protein and activity. Here we review some of the key recent advances in the field of membrane protein–lipid studies, with major emphasis on respiratory complexes, transporters, channels and G-protein coupled receptors.

## 1. Introduction

Biological membranes form barriers separating cellular or organellar contents from the external environment. These are comprised of complex mixtures of polar lipids and membrane proteins. The long-standing Fluid-Mosaic model described the structure of the membrane as a bilayer of freely laterally diffusing polar lipids forming a highly hydrophobic core and acting as solvent for the membrane proteins [1]. Whilst some features of this model still hold true, it is becoming increasingly clear that the membrane is more organised than this model suggested [2], with the presence of discrete domains or rafts in the plasma membrane acting as signalling hubs [3]. A further key feature of biological membranes is their asymmetry, with the individual monolayers that make up the bilayer having distinct lipid compositions and associated functional implications [4]. It is also becoming increasingly evident that the membrane lipids do not just act as a solvent for membrane proteins but have critical roles in their structure and function [5]. Indeed, it seems in many cases that the functional unit is a complex of membrane protein and associated lipids. An interesting study using the rhomboid protease GlpG as a model protein suggested that the presence of cavities and pockets on the external membrane-facing surfaces of a protein induce instability key for the membrane protein conformational changes. Lipid interactions in these regions do not limit the conformational flexibility of the protein but do reduce the instability associated with the presence of the cavities [6].

Until recently it has been challenging to obtain definitive information on the precise nature and role of the interactions between individual membrane proteins and membrane lipids. This has been due in part to the limitations in technologies and the fact that membrane proteins are typically solubilised from the membrane for structural and other biophysical analysis, a process using detergents that is designed to remove most if not all the interacting lipids. This has led to some controversy regarding some membrane protein structures [7]. Despite this, it has been possible to explore many important membrane protein–lipid interactions. In this review, we summarise recent advances in our understanding of the roles of lipids in a range of membrane proteins and membrane protein complexes (Table 1). 

## 2. Lipids and Respiratory Complexes

High-resolution structural studies have provided a number of insights into the role that tightly associated lipids play in the structure of membrane proteins. In virtually all cases the proteins are extracted from the membrane using detergent. However, in many cases, tightly associated lipids remain in complex with the protein even after detergent extraction and purification. If the lipids are sufficiently ordered, then they can be observed in X-ray crystal structures. One early example of lipids being clearly visible was in the crystal structure of Formate Dehydrogenase-N (Fdh-N) [50]. Each protomer of Fdh-N contains three subunits with the γ-subunit and a single transmembrane (TM) domain of the β-subunit forming the integral membrane region. FdhN crystallised as a physiological trimer with the interactions between the individual membrane domains mediated by molecules of cardiolipin (CL) forming essential interactions between the integral membrane regions of the individual protomers and clearly stabilising the oligomer. The integral membrane regions of the individual protomers have little role in this interaction, with the lipid almost entirely responsible for mediating trimer formation within the membrane. Although Fdh-N functions as a monomer, it is highly likely that trimer formation is critical for stability and thus the CL molecules are essential for formation of the physiological oligomeric state of the protein [50].

More recently, and in an exciting development, researchers have exploited a detergent-free approach using styrene maleic acid copolymer (SMA) to extract the Alternative Complex III (ACIII) from the bacterium *Flavobacterium johnsoniae* to produce SMA lipid particles (SMALPs) containing the ACIII along with native lipids [58]. The SMA approach punches holes in the membrane and surrounds the lipid and protein particles rather than disrupting the hydrophobic interactions between the membrane protein and the membrane lipids as detergents do [59]. One very appealing consequence of this mode of extraction from the membrane is that hitherto uncharacterised membrane complexes can be isolated. The researchers in this case effectively isolated a super complex of ACIII and cytochrome c oxidase. In addition, electron density assigned to 11 phospholipid (PL) molecules was also discernible in the structure in two key regions. The first region is between two of the ACIII subunits, suggesting a role for the PLs in the stability of the protein, and the second region flanks a triacylated cysteine residue in the ActB subunit, close to the site of menaquinol entry into the protein, suggesting a role for the PLs in the function of the protein [58]. Reports of lipids binding to other respiratory complexes are covered in an earlier review [60]. These include the yeast bc_1_ complex with an initial crystal structure obtained in complex with five closely associated lipid molecules [61]. Interestingly, in this case the researchers were able to alter the amount of bound lipid by changing the purification protocol, limiting the amount of time the protein spent on an ion exchange chromatography column. Protein produced with this optimised purification protocol was more active and yielded a crystal structure with an additional bound lipid [62].

A cryo-EM structure revealed lipid bound to the complete F-type ATPase from sheep [63]. Two lipid molecules are bound into the c-ring, part of the integral membrane, Fo domain, central to proton translocation. The lipids are bound into both the matrix and the intramembrane space sides of the c-ring. The e subunit of Fo, which forms part of the hook apparatus, interacts with the lipid, possibly a lysolipid, bound to the intra-membrane side of the c-ring via the C-terminal Lys residue. This lipid-mediated connection between different regions of the Fo domain is thought to increase the stability of the complex but is also likely to play a key role in ATPase function. Confirmational changes in the protein would cause movement of subunit e away from the c-ring, removing the associated lipid. It is suggested that this is an early step in full opening of the proton translocation channel [63]. 

## 3. Secondary Active Transporters

### 3.1. CitS

The structure of the CitS transporter from *Salmonella enterica* reveals the presence of a lipid, assigned as phosphatidylethanolamine (PE) on the edge of the protein [64]. It is not clear if this lipid has any specific role in CitS structure and function, but the lipid molecule is only detectable on one of the two protomers in the CitS dimer. Given that the two protomers are in different conformational states it is possible that binding of the lipid is conformationally specific. 

### 3.2. UapA and ScBOR1p

In some cases, lipids might be associated with a membrane protein but too disordered to be visible in the crystal structure. This was the case with UapA, a xanthine/uric acid transporter from *Aspergillus nidulans*, whose structure was determined as a closely associated, functionally relevant dimer with no visible lipids [65]. Native mass spectrometry (MS) analysis is proving to be a very powerful method for exploring membrane protein–lipid interactions [51,66], and this approach revealed that the UapA dimer isolates in the detergent dodecylmaltoside in complex with membrane lipids [26]. Further lipidomics analysis identified that UapA co-purifies with the membrane lipids, phosphatidylinositol (PI), phosphatidylcholine (PC) and PE. Loss of these PLs results in dissociation of the UapA dimer into the monomeric state, with the dimer recoverable through the addition of exogenous PE and/or PI. Molecular dynamics simulations predicted the location of a lipid binding site made up of three Arg residues (R287, R478, R479) at the dimer interface and on the intracellular membrane leaflet. Subsequent mutagenic and MS analyses indicated that substitution of these three Arg residues caused loss of function and resulted in protein that was almost exclusively in the monomeric form. Addition of exogenous lipid to the mutant lacking the binding site was unable to recover the dimer form, strongly indicating that binding of lipids to this site in the wild-type protein is key in the formation and maintenance of the physiological dimeric state [26]. This research highlighted that the PLs were essential for functional dimer formation and since the crystal structure of UapA is a dimer, PLs must be present in the crystals of UapA, albeit too disordered to be detectable in the final structure [65].

UapA is structurally and mechanistically related to other transporters from the solute carrier (SLC) 4 and SLC26 families, including the BOR proteins, boron transporters. The BOR protein from *Saccharomyces cerevisiae*, ScBOR1p, isolates as a monomer in both dodecyl-β-D-maltoside (DDM) and Triton X-100 [27], but lipidomics analysis reveals that it co-purifies in the presence of PI, PE, PC and phosphatidylserine (PS). As with UapA, addition of exogenous lipid to delipidated ScBOR1p causes the monomers to associate into dimers as revealed by native MS. A similar lipid binding site was predicted at the intracellular side of the dimer interface of ScBOR1p, from a model of the protein based on the crystal structure of *Arabidopsis thaliana* BOR1 [67]. Mutagenesis of the lipid binding site in ScBOR1p prevents lipid dependent dimer formation but does not abolish transport function, indicating that the dimer is not critical for function and highlighting that although there are clear similarities in the interactions between UapA and ScBOR1p and membrane lipids, the precise functional outcome of that interaction differs between the two proteins [27].

### 3.3. Monoamine Transporters, hSERT and hDAT, and the Homologue LeuT

Differences in relatively related transporters and their interactions with lipids are also seen in the serotonin (hSERT) and dopamine (hDAT) transporters, both monoamine transporters. There is strong support for both transporters being oligomeric in the native membrane, with the hSERT oligomers stabilised by phosphatidylinositol-4,5-bisphosphate (PtdIns(4,5)P_2_) binding [40]. However, whilst there is clear evidence of binding of PtdIns(4,5)P_2_ to the N-terminus of hDAT and a suggested role in amphetamine-induced dopamine efflux through the hDAT [41], stability of the hDAT dimers is independent of PtdIns(4,5)P_2_ as assessed by single molecule fluorescence microscopy before and after enzymatic depletion of PtdIns(4,5)P_2_ in the membrane [68]. Crystal structures of both the drosophila DAT [69,70] and the hSERT transporter [71] identify putative cholesterol-binding sites. Cholesterol is important for the functioning of hSERT. Recent analysis indicates that mutation of a cholesterol-binding site in hSERT or depletion of membrane cholesterol results in the transporter preferentially adopting the inward facing conformation which in turn reduces transporter activity [8,9]. Conversely, mutations in the cholesterol binding site that favour cholesterol binding cause the transporter to preferentially adopt an outward facing conformation [9]. Thus, association and dissociation of cholesterol at a key site in the protein may play an essential role in regulating hSERT activity. Given the fact that similar effects of cholesterol binding on transporter conformation are reported for hDAT [72,73], it is possible that cholesterol plays a similar regulatory role in hDAT.

A recent publication from the groups of Michael Landreh and Carol Robinson explored the concept of annular lipids that provide the hydrophobic environment essential for maintaining the overall structure of the protein [74] versus specific lipids in more detail [75]. They developed a method which exploits the fact that loosely bound, annular lipids on the surface of a protein are more prone to exchange for detergent molecules than closely associated lipid molecules. Following detergent exchange, the protein is analysed by native MS which detects changes in the abundance of bound lipid. The results of this study revealed that the bacterial presenilin homologue forms only weak interactions with annular lipids whereas LeuT, a bacterial homologue of the monoamine transporters, binds both specific lipids at a dimer interface and annular lipids on the periphery [75]. Additional analysis has indicated a role for CL in the oligomerisation of LeuT [51], a lipid also implicated in allosteric regulation of the bacterial lipid II exporter, MurJ [52].

### 3.4. The Betaine Transporter, BetP

**Figure 1:**
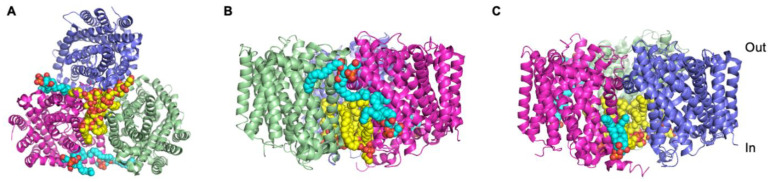
Crystal structure of the trimer of BetP in complex with the lipid (PDB: 4C7R [36]). The individual protomers are shown as green, bright pink and blue ribbons. The lipids key in trimer formation are shown in sphere representation with yellow carbon atoms. The annular lipids bound to the periphery of the protein are shown in sphere representation with cyan carbon atoms. (**A**) The protein–lipid complex is shown from the intracellular side of the membrane. (**B**,**C**) The protein–lipid complex in two different views looking through the membrane to illustrate the different locations of the peripherally bound lipids.

Although it can be difficult to obtain structural insights into transporter-lipid interactions as a result of both the loss of lipids and the poor structural resolution of the lipid molecules, it is possible. One example is the bacterial transporter, BetP, involved in osmoregulation, which crystallised in complex with lipids whose density was clearly discernible in the high resolution (2.7 Å) structure. The lipid density was assigned as eight palmitoyl-oleoyl phosphatidyl glycerol (PG) molecules [36], with seven of the PG molecules located on the intracellular side of the protein and one located on the extracellular side. Five of these lipids mediate protomer–protomer interactions within the BetP trimer (Figure 1A) playing a fundamental role in oligomer formation. The remaining three lipid molecules, including the lipid on the extracellular side of the protein, are more loosely associated with the periphery of the protein (Figure 1B,C) and are likely to be annular lipids. The lipids bound to BetP are associated with regions known to be involved in conformational changes associated with transport activity and transport regulation, strongly indicating that in addition to the quaternary structure of the protein, lipid binding is also critical for function [36].

### 3.5. The Cationic Amino Acid Transporter, GkApcT

The presence of lipid, in this case cholesterol, was essential for crystallisation of a cationic amino acid transporter homologue from the thermophilic bacterium, *Geobacillus kaustophilus,* GkApcT [76]. A cholesterol molecule sits in a pocket formed by interactions between the GkApcT and another single transmembrane domain protein, MgtS, stabilising this interaction. Clearly this is a very interesting lipid dependency since bacteria do not produce cholesterol. The authors of the study suggest that the cholesterol may be a functional replacement for a group of chemically similar lipids, called the hopanoids, found in some bacteria [77]. Whilst the precise role of the potential hopanoid interaction in vivo is not clear, it is possible that the lipid plays a role in transporter regulation as described above for cholesterol and the monoamine transporters.

### 3.6. The Major Facilitator Superfamily Sugar Transporters, LacY and XylE

An exciting new development in understanding the structural and functional implications of membrane protein–lipid interactions is hydrogen–deuterium exchange mass spectrometry (HDX-MS). HDX-MS defines the solvent accessibility of different regions of a protein by monitoring the exchange of hydrogen to deuterium; the exchange reaction depends on intrinsic protein motions [78]. H-bonding networks greatly reduce the rate and efficiency of HDX, whereas highly dynamic regions typically undergo a higher level of HDX [79]. In a recent study, Martens et al., [28] explored the conformational state of two bacterial sugar transporters, LacY and XylE, that had been purified in detergent and then reconstituted into nanodiscs incorporating a mix of PC, PG and CL or PE, PG and CL. Nanodiscs are membrane mimetic systems formed from a membrane scaffold protein, exogenous lipids and the target protein, with the scaffold protein wrapping around the complex of lipids and protein. This arrangement shields the protein from aqueous solution and produces a more native-like environment than detergent micelles whilst also allowing ready variation of the lipid composition [80,81]. The results of the study on LacY and XylE revealed that in the presence of PE both transporters were preferentially in the inward-facing conformational state. The ability to control the lipid composition surrounding isolated protein through nanodisc reconstitution is a powerful means of exploring the role of individual lipids; however, it does suffer from the disadvantage that the protein is isolated initially in detergent and then lipids added. An alternative approach is to use the SMALPs together with HDX-MS, allowing analysis of the conformational dynamics of membrane proteins encapsulated in their native membranes as described for the bacterial rhomboid protease, GlpG [82]. Here, the authors were able to alter the lipid composition at the level of the bacterial membrane by varying the expression strain or the expression temperature. They found that the conformational flexibility of the protein differed depending on the lipid composition. Such approaches have major potential for exploring the protein–lipid relationships of other classes of membrane proteins. Additionally, researchers are developing direct methods, performing MS on proteins removed directly from the membrane without the need for any extraction agents [83]. This technology is still in the early stages, but it has potential to provide information on the direct physiological interactions of membrane proteins with membrane lipids as well as protein–protein interactions.

## 4. ATP Dependent Pumps and Transporters

Lipids are also crucial for ATP dependent pumps and transporters. The Na^+^, K^+^ ATPase, for example, was crystallised in complex with a molecule of cholesterol bound between the α and β subunits [84]. The precise role of the cholesterol bound to this protein has been subject to debate, possibly due to the non-physiological conditions used for study of the effect of the lipid. However, recent research uses the addition of methyl-β-cyclodextrin to deplete cholesterol from membrane fragments while keeping all the other membrane components intact [10]. This study revealed that Na^+^, K^+^ ATPase is less active when there is a lower amount of cholesterol in the membrane and suggests that this is due to less efficient transition between different conformational states critical for the transport cycle.

Cholesterol is also important for the ATP binding cassette (ABC) transporter, PgP. PgP is an extremely important example from this family responsible for multi-drug resistance in many types of cancers [85]. The biochemical relationship between PgP and cholesterol appears to be multi-faceted, involving possible roles for cholesterol in ATPase activity, in modulating binding affinity of some transported substrates, as well as a possible direct function for PgP in cholesterol trafficking [11]. Although there is no direct structural information available for cholesterol binding to the PgP, recent MD simulations have identified several putative cholesterol binding sites and have suggested that the cholesterol cluster specifically on one side of the protein interacts with TM domain 1 [86]. The study also revealed that cholesterol flipping from one leaflet of the membrane can occur along the surface of the protein [86].

## 5. Channels

Lipids play fundamental roles in the regulation of ion channels [87]. A recent study on the two-protein domain potassium (K_2P_) channel, TRAAK, using a combination of native MS and liposome-based potassium efflux assay, allowed a detailed analysis of the lipids involved in the regulation of channel activity. The results revealed that TRAAK was activated by high affinity binding of phosphatidic acid (PA) [24].

**Figure 2:**
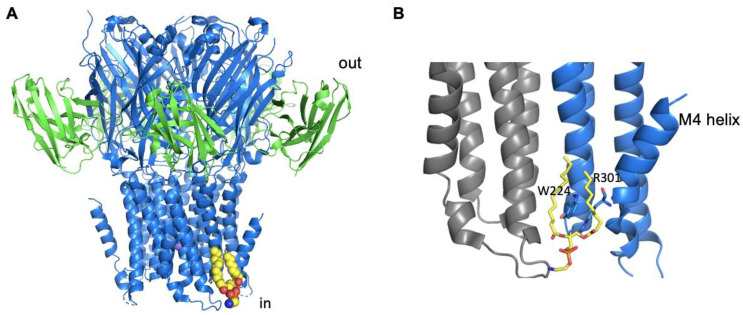
Structure of the Erwinia ligand–gated ion channel (ELIC) in complex with the lipid (PDB: 6HJX [29]). (**A**) The channel is shown in blue, and the nanobody used to facilitate crystallization is shown in green, both in ribbon representation. A bound Na^+^ ion is shown in purple, and the bound lipid is shown in yellow. (**B**) Zoomed-in view of the lipid-binding site with only two channel subunits shown (one grey and one blue) revealing the key interacting Arg and Trp residues shown in blue stick representation with blue carbon atoms and illustrating the kinked M4 helix (blue). The lipid is shown in stick representation with yellow carbon atoms.

Ligand–gated ion channels have also been analysed using MS methods revealing that the pentameric, Erwinia ligand–gated ion channel (ELIC) co-purifies in DDM with PG and PE, with a preference for PG binding [37]. The authors of this study demonstrated that addition of exogenous POPG increases the thermostability of ELIC, and they identified a likely Arg-rich lipid binding site between two subunits on the intracellular side of the TM domains. This site involves residues from TMs 1 and 4. An additional potential site on the extracellular side of the membrane was also suggested. Importantly, the researchers showed that PG binding stabilises the channel in the open, active conformation, with mutations that reduce lipid binding, increasing channel desensitization [37]. A recent structure of ELIC has conclusively identified a lipid binding site on the intracellular side of the membrane (Figure 2A), which in the structure contained a bound molecule of PE [29]. The site is similar to that predicted in the Tong et al., (2019) study and involves one of the Arg residues (Figure 2B) identified by that earlier study [37]. The structure together with MD simulations indicated that the binding of the lipid was critical for stabilising the kinked structure of the TM4 (Figure 2B), and in the absence of the stabilising influence of the lipid, this region of the protein was much more conformationally dynamic, and it is this that was suggested to be the molecular basis for the increased desensitization seen in the lipid-binding site mutants [29]. Taken together, all these findings strongly indicate that lipid binding is key to regulating ELIC gating, a feature that may also be important in some eukaryotic pentameric LGICs. Clearly, further studies are required to confirm precisely which lipid plays this role in the physiological membrane.

Duncan et al., [42] used MD simulations to build on earlier biochemical and structural studies [88,89,90] exploring lipid binding to the inward rectifier (Kir2) potassium channels. Their study confirmed the importance of PtdIns(4,5)P_2_ binding in activation of the Kir2 channels and suggested that there is cross talk between PtdIns(4,5)P_2_ binding and binding of a further phospholipid, most likely PS, to a second distinct lipid binding site.

In addition to extensive data showing that cholesterol has a key role in channel inhibition [12], these findings reveal that membrane protein–lipid interactions can be very complex indeed.

Lipids have been revealed in the structures of Transient Receptor Potential (TRP) channels, nonselective cation channels sensitive to a range of environmental changes including alterations in temperature and pressure [91]. A recent example is the TRP from the alga, *Chlamydomonas reinhardtii*, whose tetrameric structure revealed the presence of three lipid molecules per protomer [43]. These were assigned as two PtdIns(4,5)P_2_ molecules and one PC molecule binding to three distinct sites. PtdIns(4,5)P_2_ binding at one site, site 2, is key for channel opening, and removal of that binding site results in a loss of PtdIns(4,5)P_2_-induced channel activation. The second PtdIns(4,5)P_2_ binding site is similar to the vanilloid binding site, occupied by a PtdIns(4,5)P_2_ molecule in the structure of the mammalian TRPV1 channel. In the case of the TRPV1 channel, it is thought that the bound PtdIns(4,5)P_2_ stabilises the inactive state of the channel, which is activated upon binding of a specific ligand into the vanilloid binding site, displacing the bound PtdIns(4,5)P_2_ molecule [44]. Whilst it is not clear precisely what role the equivalent site plays in the *C. reinhardtii* TRP channel, it seems probable that membrane lipids can act as allosteric modulators of these proteins. This is supported by structures of the temperature sensitive, mouse TRPV3 channel which revealed a lipid, likely to be a phospholipid, bound to the vanilloid binding site in the closed state but not in the open state of the protein. Loss of the lipid at the phospholipid binding site is postulated to be key to transition from the closed to the sensitized and ultimately the open state of the channel upon heat-induced activation [92].

## 6. G-Protein Coupled Receptors

**Figure 3:**
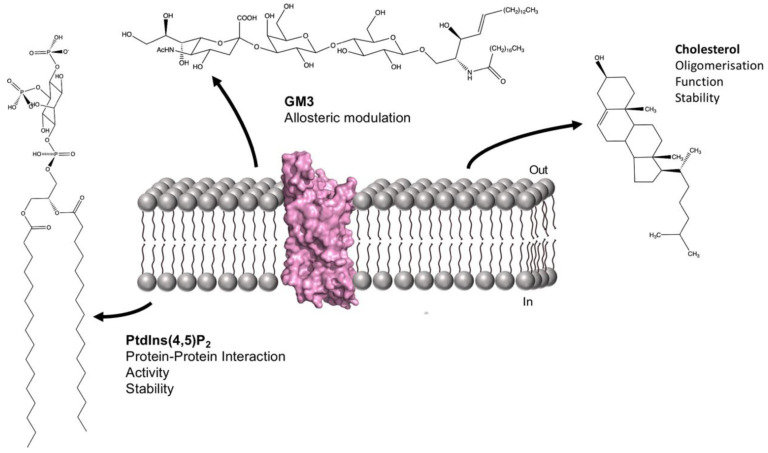
Schematic illustrating some key GPCR-lipid interactions. The structure of the A_2A_R (PDB: 5UEN [93]) is shown in space filling representation embedded in the lipid membrane. The chemical structures of GM3, cholesterol and PtdIns(4,5)P_2_ are shown together with details of their known effects on GPCR structure and function.

G-protein coupled receptors (GPCRs) are crucial for cellular responses to a range of bioactive molecules including hormones, neurotransmitters and many drugs. As a result of their biological and pharmacological importance, they have been extensively studied. A vast body of research has accumulated on the roles of lipids in GPCR structure and function. Figure 3 illustrates some of the membrane lipids and their roles. Given the nature of the current state of the art with respect to GPCR-lipid interactions, the following sections have been organised mainly according to lipid rather than protein.

### 6.1. GPCRs and Cholesterol

It has long been known that cholesterol has a key role in GPCR structure and function [94]. Cholesterol directly affects the ligand–binding ability of several GPCRs, including the subtype 2 galanin receptor and the serotonin 1A receptor [95], and there is evidence that cholesterol also plays a role in GPCR signalling, for example, increasing basal activity of the cannabinoid 2 receptor [14]. In the recent study of the class F GPCR, Smoothened, cholesterol is revealed to traffic through a channel in the receptor and play a fundamental role in receptor activation [13].

In other cases, the more indirect effects of cholesterol on the biophysical properties of the membrane appear to be important [96]. Cholesterol is important for the stability of receptors, as supported by a raft of different GPCR structures reviewed in Gimpl [16]. Cholesterol binding was observed in a groove created by TMs 1-4 in a high-resolution structure of the β_2_-adrenergic receptor [97], leading to the identification of the Cholesterol Consensus Motif (CCM) found in multiple receptors. Interestingly many subsequent GPCR structures exhibit cholesterol binding at other sites and not the CCM, even when a CCM–binding site is present [16]. In addition to the CCM, a range of other cholesterol binding motifs are found in GPCRs which may accommodate these lipid molecules. The wide variety of cholesterol–binding sites seen across the different GPCR structures indicates that cholesterol binds promiscuously across the surface of the proteins, with specificity conferred by individual conformational states and the individual requirements of a given receptor [16]. Further computational analysis of a range of X-ray and cryo-EM structures indicates that cholesterol binds to a number of regions of GPCRs and that these sites are not characterised by specific motifs [98]. This is supported by a recent MD simulation study on 28 individual GPCR structures, including some active and inactive states of the same receptor [18]. In this case, the study revealed that the numbers and sites of the binding of cholesterol molecules differ between both different receptors and alternate conformational states of the same receptor.

A nice example of receptor–specific interactions with cholesterol is provided by the recent structure of the Oxytocin receptor (OTR) [17] which was crystallised in complex with a molecule of cholesterol bound to a site between helices 4 and 5. This study also revealed that mutation of residues involved in cholesterol binding reduced the stability of the OTR in the presence of exogenous cholesterol hemisuccinate (CHS), compared to a receptor construct with the cholesterol binding site intact. Furthermore, mutation of these cholesterol binding residues substantially reduced agonist and antagonist binding compared to the WT OTR. Given the proximity between the cholesterol–binding site and the ligand binding site, it is suggested that cholesterol binding is crucial for maintaining the optimal arrangement of amino acid residues within the ligand–binding site [17]. Further research on the OTR supports the fact that cholesterol is key for high affinity ligand binding but also that the act of ligand binding stabilises the interaction between the receptor and the bound cholesterol [99]. It is postulated that ligand binding may induce dimer formation, thus burying one or more cholesterol molecules at the dimer interface.

There is much evidence that cholesterol plays a role in GPCR oligomerisation. Initial indications of this came from the first structure of the β_2_-adrenergic receptor β_2_AR, which revealed a role for cholesterol in mediating dimer formation through a TM1 and TM7 interface [19]. A range of subsequent studies have provided supporting evidence of cholesterol having a role in both receptor homo-oligomerisation [20,21,22] and hetero-oligomerisation [23] of GPCRs. In the case of the β_2_AR receptor, the cholesterol interacts with the palmitoyl group post-translationally added to a Cys residues in the C-terminal region of the protein [19]. Such an interaction has also been suggested for the μ-opioid receptor. In this case, removing the palmitoylation site reduced cholesterol association with the receptor and this decreased receptor signalling. Cholesterol depletion also reduced receptor signalling [15]. However, subsequent MD simulation analysis indicates that this cholesterol–palmitoyl interaction seems to occur preferentially in the inactive form of the receptor, and in the case of μ-opioid receptor, cholesterol does not appear to have a clear role in dimerization [100].

A structure of the yeast GPCR, Ste2, in the dimeric form and in complex with 2 cognate heterotrimeric G-proteins, has recently been reported [101]. In this structure, density assigned to 6 cholesteryl hemisuccinate (CHS) molecules was observed close to the dimer interface. These were assigned as CHS, since this sterol was added to the buffers during isolation of the receptor. However, it is possible that some if not all of these are native ergosterol molecules with a role in stabilising the dimer interface and carried through the solubilisation and purification of the receptor.

There is also some evidence from MD simulations that cholesterol and phospholipid compete for binding at some receptor sites, with phospholipids shown [102] and suggested to [39] displace cholesterol bound to the adenosine 2A receptor (A_2A_R). Given that lipid binding is stronger when the receptor is in the active state and in complex with G-protein, a combination of specific bound lipids at defined sites is likely to play a role in regulating receptor activity [102].

### 6.2. GPCRs and Phospholipids

Many studies have revealed the contribution of phospholipids in modulating the stability and activity of GPCRs, as well as the selectivity of G-protein coupling. Dawaliby and colleagues demonstrated that DOPE induced a significantly reduced affinity for agonist binding to the β_2_AR reconstituted into high–density lipoparticles compared to DOPG [30]. In contrast, β_2_AR reconstituted in DOPE lipoparticles exhibited higher binding affinity for the antagonist compared to DOPG and DOPI. Further experiments revealed that β_2_AR preferentially co-purifies with PG, and that PG provides the most favourable environment for binding to a G-protein mimetic [30], indicating that in the case of this receptor, negatively charged lipids are important for receptor activation. These findings indicated that PLs modulate receptor activity by stabilising different specific receptor conformations, and this is further supported by MD simulations on the A_2A_R which indicate that PG together with ligand binding induces the active form of the receptor, while a combination of ligand and PC is unable to induce the active from of the receptor [39]. An additional MD–based survey of 28 GPCR structures, from different classes, identified PIP lipids as forming the closest interactions with the receptors, although the precise molecular basis of the interactions seems to differ for individual receptors [18]. The important role of PIP lipids is underlined by a study that utilised a combination of mass spectrometry analyses and MD simulations revealing that PtdIns(4,5)P_2_ binds to positively charged residues on the intracellular side of class A GPCRs, stabilising the active states of the receptors [25]. Similar results have been obtained for the GSHR, ghrelin receptor, with FRET analysis using labelled PtdIns(4,5)P_2_ and labelled ghrelin receptor revealing that PtdIns(4,5)P_2_ binds preferentially to the active form of the receptor [45].

MD studies on the neurotensin receptor (NTSR1), revealed that POPC promoted much greater dimer formation than physiological–simulated membranes based on brain polar lipids. The dimer interfaces adopted in POPC involved TMs 1, 5 and 6 in both symmetrical and asymmetrical protomer arrangements [34]. In contrast, in the brain polar lipid membrane, the NTS1 dimers form with a range of different interfaces involving TMs 1-6, in agreement with experimental studies on the same receptor [103]. This MD study also highlighted that different lipids stabilise different dimer conformations, with, for example, PS stabilising a symmetrical dimer involving TMs 3 and 4 of each protomer [34]. This dimer interaction interface leaves TMs 5 and 6 free to interact with the G-protein, suggesting that PS binding at the dimerization interface may be more favourable for the active forms of the receptor than PC. Since dimerization/oligomerisation interfaces are suggested to be partially dependent on protomer conformation [100,104,105], phospholipids can favour receptor–receptor contacts at particular interfaces by binding favourably to certain conformations. This suggests that by stabilising a certain receptor oligomeric state, phospholipids may modulate receptor activity.

Whilst it is clear that phospholipid head groups are important, there is also support for the fact that the acyl tails of phospholipids play a role in GPCR function and organisation. A recent study explored the effect that lipids with long (22 C), polyunsaturated tails derived from docosahexaenoic acid (DHA) have on the A_2A_R. The findings revealed that the DHA–derived lipids resulted in increased populations of A_2A_R in the active conformation and greater G-protein coupling compared to lipids with shorter acyl tails but the same head group [53]. A number of MD studies have supported a role for DHA-containing unsaturated phospholipids as these order around the NTS1 [34] and drive A_2A_R to partition to lipid rafts [55]. A very recent MD study indicated that solvation of A_2A_R by unsaturated acyl chains is thermodynamically more favourable than saturated acyl chains, shifting the equilibrium towards active conformers [54]. In contrast, saturated acyl tails, which form part of the lipid raft domains from which DHA was excluded, allow formation of functional dimeric rhodopsin [57].

Phospholipids also exert an influence over GPCRs by changing the bulk membrane properties [106]. For example, unsaturated chains are known to cause hydrophobic mismatch between receptors and the membrane [107]. This can drive non-specific receptor oligomerisation [34,56], as a means of combatting the free energy penalty caused by the mismatch [108]. Mismatch–driven oligomerisation may also partially be a result of receptor activation [96]. However, this does not necessarily mean higher-order structures driven by mismatch are not functionally important; mismatch is suggested to aid partitioning of rhodopsin to lipid domains in central regions of the disc membrane, thus allowing efficient coupling to G-proteins [57].

### 6.3. GPCR Complexes and Phospholipids

**Figure 4:**
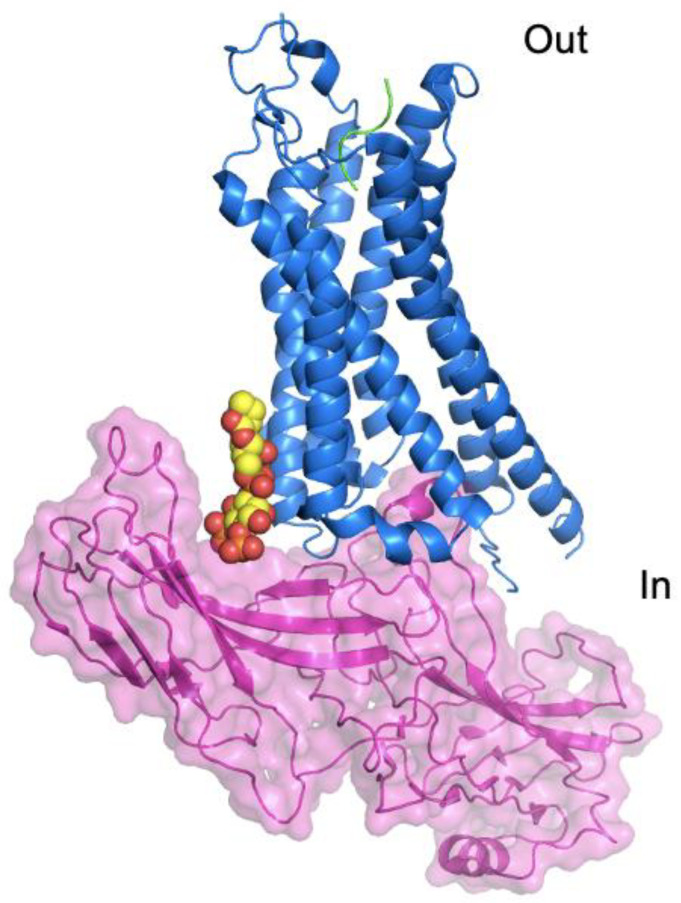
Structure of the NTSR + β-arrestin complex (PDB: 6UP7 [46]). The NTSR is shown in blue ribbon representation with the bound shown in green. The β-arrestin is shown in pink transparent surface representation. The PtdIns(4,5)P_2_ in contact with both the receptor and the β-arrestin is shown in space filling representation with yellow C atoms.

There is increasing evidence that the lipid bilayer plays a key role in interactions between GPCRs and key binding partners. β-arrestin binding is responsible for both desensitization and internalisation of GPCRs and G-protein independent intracellular signalling [109,110]. The recent structure of an engineered form of muscarinic M2 receptor in complex with β-arrestin 1 obtained in nanodiscs (comprised of POPC, POPG and the membrane scaffold protein, MSP1D1E3) revealed that β-arrestin 1 interacted with the nanodisc encapsulated lipids as well as the receptor [35]. Additional data suggested that this β-arrestin 1-lipid interaction might be crucial for physiological receptor-β-arrestin 1 affinity by providing an additional source of complex stabilisation. The β-arrestin 1-lipid interaction is also important for β-arrestin 1 function in terms of modulating agonist binding to the receptor and receptor desensitization and internalisation [35]. Further support for lipids playing a role in receptor-β-arrestin binding has come from an additional cryo-EM complex structure; in this case, the NTSR1 in complex with a modified form of β-arrestin 1 [46]. The structure revealed that a molecule of PtdIns(4,5)P_2_ mediates interactions between the receptor and the β-arrestin (Figure 4). Mutating the PtdIns(4,5)P_2_ binding site in β–arrestin results in reduced β-arrestin binding to the receptor. These findings strongly suggest that the lipid has a role in the recruitment of β-arrestin and subsequent stability of the receptor-β-arrestin complex [46]. It is possible that there is receptor–dependent variability in the precise nature of the interactions with β-arrestin but the variability described in just these two examples may also reflect differences in sample preparation prior to structural analysis. However, it is clear that lipids have the potential to modulate receptor function at the level of the GPCR itself as well as through direct interaction with GPCR effector molecules.

Lipids are also suggested to play a role in interactions between GPCRs and G-proteins. The MS analysis study by Yen et al., revealed that PtdIns(4,5)P_2_ bound to the intracellular regions of GPCRs stabilises the active conformation of the receptor and increases interactions with the G-protein [25]. In contrast, fluorescence spectroscopy and mutational analysis determined that PG/PS lipids diminish coupling of G_i1_ and G_i3_ to β_2_AR under conditions of low Ca^2+^, likely as a result of repulsion between the negative charges of PG/PS and G_i_ [31]_._ Higher levels of β_2_AR-G_i3_ coupling were observed in the presence of PE/PC lipids, with Ca^2+^ mediating the G_i3_-PE/PC interaction [31]. This suggests that phospholipids not only aid discrimination between G protein types by increasing the population of a conformer that couples to a specific G protein but also by directly promoting GPCR-G-protein interactions by mediating key electrostatic interactions.

Using microscale thermophoresis, Zhang et al., [38] suggested that NTSR1 coupling to Gα_i_ was mediated by PG at the interaction interface, in contrast with earlier studies which suggested NTSR1 coupling to Gα_i_ is enhanced by PE-rich membranes [32]. Yet further studies using native MS revealed that the NTSR1 purifies in the presence of PS, PA and PIP species and shows preferential binding to these lipids when added exogenously compared to PC [25]. This study revealed no detectable PG binding, with the authors suggesting that any effects of PG could be a result of alterations in the local membrane charge around the receptor. Among the lipids tested, PtdIns(4,5)P_2_ bound most effectively to the NTSR1, as well as the β_1_AR and the A_2A_R, and mutagenesis of a predicted PtdIns(4,5)P_2_ binding site formed on the intracellular side of the protein and involving principally positively charged residues in TM4 resulted in loss of PtdIns(4,5)P_2_ binding [25]. Further analysis revealed that NTSR1-G-protein coupling was increased in the presence of PtdIns(4,5)P_2_ and given the location of PtdIns(4,5)P_2_ binding to the receptor, this is likely to be the result of the lipid mediating interactions between the receptor and the G-protein. Since similar results were obtained with the β_1_AR and the A_2A_R, this suggests that the role of PtdIns(4,5)P_2_ in G-protein coupling is common to other Class A GPCRs [25,102].

MD simulations indicate that PtdIns(4,5)P_2_ interacts with the glucagon receptor, GCGR, a class B receptor, in some sites conserved with those in class A GPCRs [47]. However, no PtdIns(4,5)P_2_ binding was detectable at the TM3/ICL2 site shown to be important for class A receptor G-protein recruitment. These findings suggest that the roles PtdIns(4,5)P_2_ plays in class B receptor function are distinct from those of class A receptors [47].

### 6.4. GPCRs and Sphingolipids and Glycolipids

Use of a mycotoxin known to reduce sphingolipid content reduces the amount of cell surface localised 5HT-1A receptor [111], in line with earlier results indicating the same treatment reduced specific ligand binding and associated downstream cAMP signalling for this receptor [48]. In contrast, similar studies on the angiotensin II type 1A receptor and the bitter taste receptor, T2R14, indicated no change in receptor signalling as a result of sphingomyelin depletion [112].

Gangliosides (GMs) are a type of glycosphingolipid found mostly on the membrane outer leaflet [113]. Coarse-grain MD simulations proposed that GM1 binds to an identified and conserved “sphingolipid binding domain” on extracellular loop (ECL) 1 of the 5HT-1A receptor and modulates ligand binding [49]. MD simulations also indicate that GM3 binds to ECL1-3 and extracellular portions of TMs of both the class B glucagon receptor (GCGR) [47] and the class A A_2A_R [102] through basic and aromatic residues. In the case of GCGR, GM3 binding to the extracellular domain (ECD, also responsible for ligand binding) affects the conformational dynamics of this region of the receptor, thus potentially acting as an allosteric modulator affecting the ability of the receptor to bind ligands [47].

Given the high percentage of sphingolipids and glycosphingolipids in lipid rafts, these and other findings support an important relationship between lipid rafts and GPCRs [114]. However, other MD simulations have indicated that while GMs were enriched around GPCRs, sphingomyelin was depleted around the 28 GPCR structures they probed, relative to the bulk membrane, suggesting that some sphingolipid species play little role in stability, function or organisation of these receptors [18]. However, it is also possible that the differences in the simulation methodology used are responsible for some of the different results obtained. The lack of high–resolution GPCR structures in complex with GMs and SMs currently limit our overall understanding of the precise nature of these interactions.

## 7. Membrane Lipids and Other Membrane Proteins

Lysosome–associated protein transmembrane 4B (LAPTMB) is responsible for mediating traffic of amino acid transporters to lysosomes under conditions of high nutrient availability. Experimental and MD simulation data indicated that a lipid binding site in TM3 specific for ceramide is crucial for correct dimerization of LAPTMB and the amino acid transporter proteins [115]. EPR and mutagenesis-based analysis of Annexin B12 indicates that oligomerisation of the protein is highly dependent on membrane lipids [116], in addition to protein–protein interactions. However, the precise lipids that mediate the oligomer formation have yet to be identified. Connexins are integral membrane proteins that associate to form gap junctions between cells and allow the passage of information and small molecules from one cell to another. A likely lipid binding site was recently identified in the cryo-EM structure of the Cx31.3 connexin hemi-channel obtained at a resolution of 2.4 Å. This binding site located within the pore cavity is suggested to have a role in connexin hemi-channel assembly [33]. Density close to this binding site was assigned as a PE molecule, which had been extracted from the membrane and copurified with the hemi-channel [33]. Further work on the Connexin-46/50 full cell–cell junction focused on the protein obtained in DMPC-containing nanodiscs. The integral membrane domains on the extracellular side of the two hemi-channels are stabilised by extensive clusters of lipid molecules. These ordered lipid molecules extend further out from the protein than is typical for lipids forming specific interactions, a finding further supported by MD simulations [117]. This study raises the possibility that formation of the full cell–cell junction induces local order in the membrane environment [117].

## 8. Conclusions

As summarised in the current review, insights into membrane protein–lipid interactions are increasing rapidly. The development of novel, non-detergent-based membrane mimetic systems and the use of lipid reconstitution approaches such as nanodiscs for mass spectrometry and electron microscopy sample preparation pave the way for a more physiologically relevant understanding of the interactions between membrane proteins and membrane lipids. In addition, it is clear that the ability to probe potential membrane protein–lipid interactions in silico using Molecular Dynamics simulations where the researcher has unparalleled control of the membrane lipid composition provides an excellent means of providing context to experimental findings and forms the basis for further studies. We are just starting to unpick these complex and important molecular relationships and there is much more to do.

## Figures and Tables

**Table 1:** Summary of the lipid interactions covered in this manuscript.

		Membrane Protein					
		Respiratory Complexes	2° Active Transporters	ATP-Dependent Pumps/Transporters	Channels	GPCRs	Other
Lipid entity	Cholesterol		Activity regulation [8,9]	Activity and changes in conformation [10], Modulation of substrate binding affinity [11]	Channel inhibition [12]	Activation [13], Signalling [14,15], Stability [16,17], Allosteric regulation [18], Lower affinity ligand binding [17], Oligomerisation [19,20,21,22,23]	
	PA				Activity [24]	Stabilisation [25]	
	PE		Dimer formation and function [26], Dimer formation [27], conformer stability [28]		Conformer stabilisation and channel desensitisation [29]	Agonist and antagonist binding affinities [30], Increase G protein coupling [31,32]	Protein assembly [33]
	PC					Dimerisation [34], β-arrestin interaction and function [35], Increase G protein coupling [31]	
	PG		Oligomerisation and function [36]		Conformer stabilisation and channel desensitisation [37]	Increase G protein coupling [30,38], Active conformer stability [39], β-arrestin interaction and function [35], Decrease G protein coupling [31]	
	PS		Dimer formation [27],			Stabilisation [25], Dimerisation [34], Decrease G protein coupling [31]	
	PI		Dimer formation and function [26], Possible stabilisation [27]				
	PtdIns(4,5)P_2_		Possible dimer stabilisation [40,41],		Activation [42,43], Inactive conformer stability [44]	Active conformer stability [25,45], Increase G protein interaction [25], β-arrestin interaction and complex stability [46], Important for G protein recruitment [47]	
	Glycolipids and sphingolipids					Ligand binding [48,49], Signalling [48], Allostery [47]	
	Cardiolipin	Oligomer stabilisation [50]	Oligomerisation [51], Allosteric regulation [52]				
	DHA and unsaturated tails					Active conformer stability [53,54], Increase G protein coupling [53], Partitioning to lipid rafts [55], Oligomerisation [34,56]	
	Saturated tails					Dimerisation and function [57]	
